# Best Educational Techniques in High-Fidelity Simulation According to Nursing Students—Adaptation and Validation of the Educational Practices Questionnaire (EPQ)

**DOI:** 10.3390/ijerph192214688

**Published:** 2022-11-09

**Authors:** Katarzyna Zalewska, Danuta Zarzycka

**Affiliations:** 1 Carpathian State College in Krosno, 38-400 Krosno, Poland; 2 Department of Pediatric Nursing, Medical University of Lublin, 20-059 Lublin, Poland

**Keywords:** simulation, education, Educational Practices Questionnaire (EPQ), validation

## Abstract

The purpose of this study was to evaluate the best educational techniques used during high-fidelity simulations in training nursing students and to introduce the Polish version of the Educational Practices Questionnaire (EPQ) scale after its cultural adaptation and determination of its psychometric properties. The research group was composed of 361 second- and third-year nursing students in the licentiate program. The Cronbach’s alpha reliability coefficients for the adapted tool were 0.90 for the EPQ-PO (presence of educational techniques) subscale and 0.93 for the EPQ-IO subscale (importance of educational techniques). Additionally, the model fit rates in the CFA and EFA (as indicators of theoretical validity) proved to be high enough for the tool to be successfully used in scientific research. Preliminary results are also presented; the mean value of the response for the entire EPQ scale for both the PO and IO sections was M = 4.3, SD ± 0.90. The students in the study rated the opportunity for collaboration with other students and for working jointly on a given clinical situation very highly at M = 4.5, SD ± 0.70. The analysis of the scores of the individual scales and subscales of the EPQ showed statistically significant differences in results obtained for such variables as gender, place of residence, and year of studies.

## 1. Introduction

Several definitions of “simulation” can be found in the literature on the subject. One of the most common and best-known is that of D. Gaby, which is as follows: “Simulation is a technique—not a technology—to replace or amplify real experiences with guided experiences that evoke or replicate substantial aspects of the real world in a fully interactive manner” [1] (pp. 1–10). Another definition of simulation, significant to this study, was introduced by P. Jeffries, a nurse involved in simulations in nursing for many years. According to her, “Simulation is defined as an activity that mimics the reality of the clinical with the goal of presenting procedures, making decisions, and critical thinking” [2] (pp. 96–103). The Society for Simulation Healthcare (SSiH), in the latest edition of its dictionary Health Care Simulation Dictionary, Second Edition, defines simulation as a technique that creates a situation or environment that allows individuals to experience the depiction of a real event for purposes of practicing, learning, evaluating, or testing or to help understand systems or human efforts [3]. The abovementioned definitions bring together several aspects: simulation as a didactic method in which, in non-real conditions, the goal is to reproduce reality as accurately as possible in order to practice practical skills, as well as critical thinking and working in a group. It is a method that places high demands on both teachers and the students who use it. The goal of simulation in the education of nursing students is to imitate various areas of activity in a manner adapted to learning objectives—i.e., with low or intermediate fidelity—and ending with team participation in implementing the simulation scenario in high-fidelity classes [4,5]. When implementing scenarios, students play various roles in an environment made as closely similar to reality as possible [6,7]. In addition to forming the skills associated with playing a given role, it is extremely important to form the ability to work in a group, solve problems, and make decisions together [5,8].

### 1.1. Background

In its 2013 publication “Transforming and scaling up health professionals’ education and training” [9], the World Health Organization (WHO) recommends the use of simulation in educating students as a method to help them acquire skills and improve learning outcomes. It also suggests the possibility of using various types of simulators to provide the opportunity to perform a given procedure several times in safe conditions, which is not always possible in real-life conditions. It is worth ensuring that classes are conducted using appropriate equipment and by professionally prepared people; i.e., instructors or teachers [10]. It is essential in the education of students that their newly acquired theoretical knowledge be combined with practice. Therefore, simulation is a place where the student can learn practical skills, incorporating them into this his/her newly acquired theoretical knowledge, in a safe environment and without harm to patients [11,12,13,14].

Implementation of the high-fidelity simulation method in nursing education in Poland is linked to the development of a network of simulation centers. Its systematic development and the pursuit of the highest quality of education have led to a need for empirical testing of simulation results using tools, and these results constitute a valuable source of information on the various aspects evaluated during simulation classes [5].

Currently, there is no standardized research tool available in Poland that can be used to assess the educational techniques used by instructors during simulation classes. In the INACSL Standards of Best Practice: Simulation Participant Evaluation [15], there is a provision for the use of formative assessment tools associated with participants’ personal and professional development, supporting their pursuit of goals, or of summative assessment regarding measuring outcomes or achieving goals at a certain point in time. The INACSL Standards of Best Practice: Simulation Participant Evaluation also lists evaluation of critical situations. In summarizing the above assessment methods, one of the criteria of these standards is the use of a standardized tool previously tested on similar populations.

### 1.2. Framework

The basis for the development, implementation, planning, and evaluation of simulation activities in nursing are the so-called simulation frameworks, first published in 2005 [16,17] and created by P. Jeffries, which after numerous changes eventually became known as the NLN Jeffries simulation theory. The six components that create the abovementioned simulation frameworks are context, background, design, educational practices, simulation experience, and outcomes [2]. Teachers should consider each of these elements when designing curricula that incorporate high-fidelity simulation. This will ensure that students achieve the best possible learning outcomes [18,19]. In healthcare simulation, “high fidelity” refers to the highest representation of reality, including all details related to the environment or the patient’s condition, among others objects [3]. These classes use a high-fidelity simulator (human patient simulator (HPS)) that fully reflects the patient’s condition according to his or her health or life status. Realism is enhanced by adapting the simulator to the real environment by dressing it appropriately (e.g., wig, hospital clothing, etc.) [20]. Similarly, during classes conducted using the indirect-fidelity simulation method, simulators replicating basic vital functions (e.g., heart rate, respiration) are used, with which skills related to the nursing profession are honed [21]. Finally, low-fidelity simulation classes use trainers with whom learners acquire and perfect basic technical skills (e.g., blood collection or intramuscular injection) [3]. Classes delivered using this method are the least realistic simulations of the patient’s condition or environment and are also the cheapest [22].

### 1.3. Aim

The aim of this study was to assess the best educational techniques used in high-fidelity simulation in training nursing students and to present the cultural adaptations of and psychometric values in the Polish version of the Educational Practices Questionnaire (EPQ). So far, successful attempts at validating and adapting the EPQ scale have been carried out in several countries around the world: namely, in Oman, the United States, Hong Kong, Norway, Turkey, and Spain [23,24,25,26,27,28,29].

## 2. Materials and Methods

### 2.1. Sample and Settings

The study began in October 2019 in two academic centers in Poland, one of which was a university and the other a vocational institution of higher learning. A statistically specified number of correctly completed questionnaires, using the pen-and-paper personal interview (PAPI) method, were collected in June 2020. The study group consisted of 361 students in the field of nursing. Second- and third-year undergraduate students (first degree) were included in the study. Master’s students, on the other hand, use the high-fidelity simulation method in their training process to the extent decided by the university. There are no uniform guidelines in this regard, unlike for undergraduate education. Therefore, in order to ensure the comparability of results, it was decided to carry out the research project among undergraduate students, although the project has development potential and will certainly be continued in diverse educational environments. The majority of the respondents were women (96.4%), which reflects the country’s gender structure in the field of nursing. The average age of respondents was M = 21.78, SD ± 3.19. The age of 21 years was set as a cut-off point due to the following: (1) students under 21 years of age were continuing their studies immediately after secondary school and were part of the study group (51%) consisting of second-year students; and (2) students over 21 years of age were students undertaking studies to complete their qualifications and were part of the study group (49%) consisting of third-year students. The students participating in the study completed a paper version of the scale, without the presence of third parties, after completing high-fidelity simulation classes; this took about five minutes. Table 1 presents the characteristics of the study group.

### 2.2. Measures

The Educational Practices Questionnaire (EPQ) test scale consists of sixteen questions divided into the following subscales: Active Learning (items 1–10), Collaboration (items 11 and 12), Diverse Learning Methods (items 13 and 14), and High Expectations (items 15 and 16). Furthermore, the scale is divided into two parts. One of these concerns the presence of statements given during simulation classes (EPQ-PO), and the other one concerns their validity, in the student’s opinion, during the classes (EPQ-IO). The Cronbach’s alpha coefficients were 0.86 for the EPQ-PO and 0.91 for the EPQ-IO [16]. The scores were computed by adding up the responses; higher scores mean greater recognition of educational best practices in simulations [23]. The consent (non-commercial use) for the use of the tested tool was obtained according to National League of Nursing instructions for use of surveys and research instruments [30].

### 2.3. Cross-Cultural Adaptation Process

The cultural adaptation and psychometric validation of the tool being presented were undertaken according to the schema of the World Health Organization [31]. They consisted of the following stages:

1. Stage I: obtaining the consent of the authors for cultural adaptation and psychometric validation of the EPQ scale;
2. Stage II: performing a translation of the scale with two independent translators from English to Polish (English forward translation);
3. Stage III: development of the Polish version of the EPQ scale by a panel of experts;
4. Stage IV: conducting a “backward” translation from Polish to English and comparing the two versions of the scale (English back translation);
5. Stage V: establishing the final version of the EPQ scale;
6. Stage VI: assessing the psychometric properties of the final version of the EPQ scale.

### 2.4. Ethical Considerations

The study was approved by the Bioethics Commission of Medical University of Lublin (KE-0254/348/2018). Consent for carrying out the study was granted by the individuals upon whom the study was conducted. During the course of the research, participants gave signed consent for informed and voluntary participation in this study. The questionnaire dealt with basic questions such as age, gender, year of studies, class topic, and the role played in the scenario. All of these data were anonymous. In addition, the study was conducted in accordance with the Declaration of Helsinki.

### 2.5. Reliability

The Cronbach’s alpha coefficient and item-total correlation were used to assess the internal consistency and reliability of the presented scale. Values above 0.70 were adopted as the acceptable values for the Cronbach’s alpha coefficient [32]. For item-total correlation, values equal to or greater than 0.40 were considered acceptable [33].

### 2.6. Psychometric Validity

Confirmatory factor analysis (CFA) was used to determine the validity of the construct. The following indicators were used to evaluate the fitness of the model for determining validity: the standardized root mean square residual (SRMR), root mean square error of approximation (RMSEA), weighted root mean square residual (WRMR), comparative fit index (CFI), normed fit index (NFI), and the Tucker–Lewis index (TLI) [34]. The following values were adopted as permissible for the individual components of the model: SRMR values greater than 0.05 represented a perfect match for the model and results greater than 0.10 a good match for the model; the WRMR, CFI, NFI, TFI, and TLI ranged from 0 to 1, where 1 meant a perfect fit.

Exploratory factor analysis (EFA) was conducted to determine the factor structure. It is worth mentioning that relations between construct and variables are significant, as they make it possible to determine the direction and structure of the relationship between construct and measures [35]. Discussion is ongoing among many researchers as to the size of (research) groups; Ref. [36] recommends in his work that the size of a group under study should be greater than 100 [36], while de Winter et al. (2016) demonstrates that credible and acceptable EFA results can be obtained from groups of fewer than 50 [37]. Based on the above evidence, the authors of this study concluded that their sample size was adequate. Bartlett’s test of sphericity was used to verify the homogeneity of variance of the compared series, without using a correlation matrix, with *p* = 0.05. Additionally, the adequacy of the sample was determined using the Kaiser–Meyer–Olkin (KMO) criterion, with a range from 0 to 1 and values above 0.60 considered acceptable [38,39].

### 2.7. Data Analysis

The analysis was performed with GNU R software—R version 4.0.4 (R Core Team. R: A Language and Environment for Statistical Computing; R Foundation for Statistical Computing: Vienna, Austria, 2016).

## 3. Results

The Cronbach’s alpha reliability coefficients for the tested tool were 0.90 for the EPQ-PO subscale and 0.93 for the EPQ-IO subscale. When removing a given item from the scale, no increase in the Cronbach’s alpha coefficient was noted; hence, analysis of items based on correlation led the researchers to conclude that no items needed to be excluded from the scale. In the context of scientific evidence, these are very satisfactory results. The analysis of the items is presented in Table 2. The item-total correlation ranged from 0.45 to 0.65 for the EPQ-PO and from 0.60 to 0.69 for the EPQ-IO. The strongest relationship to the summary result among the individual questions of the EPQ-PO subscale was obtained for the fifteenth question: *r* = 0.65. On the other hand, the weakest correlation factor for the EPQ-PO subscale was the second question: *r* = 0.45. For the EPQ-IO subscale, the strongest relationships between the questions and the summary result were in the 9th, 13th, and 15th questions: *r* = 0.69. The weakest correlation for the EPQ-IO subscale was in the sixth question: *r* = 0.60.

All indicators obtained in the confirmatory factor analysis (CFA) and used to determine validity are presented in Table 3. They attained the desired values and indicated a good fit between the model and the data. The tool, therefore, can be successfully used in scientific research.

Before conducting exploratory factor analysis (EFA), the Kaiser–Meyer–Olkin (KMO) test and Bartlett’s sphericity test were performed. As shown in Table 4, the values for the KMO criterion were fully acceptable for both the EPQ-PO and EPQ-IO. Additionally, the presented scale achieved statistical significance in Bartlett’s sphericity test, both for the EPQ-PO subscale (χ^2^ = 2104.67; *p* ≤ 0.001) and for the subscale related to the validity of these features (χ^2^ = 2875.62; *p* ≤ 0.001). When developing the EFA model, a four-factor model was obtained for both the EPQ-PO and EPQ-IO subscales. The loads obtained in this EFA factor analysis for the EPQ-PO subscale explained 50.60% of the variance, and those for the EPQ-IO subscale explained 58.40%. The CAF model fit for the EPQ-PO subscale was 0.48; for the EPQ-IO subscale, it was CAF 0.50. When analyzing the loads received for individual items, it can be seen that most of them achieved a value above 0.40. Nevertheless, the EFA model did not fully justify the received loads for individual items that were loaded onto several factors (Table 5).

### 3.1. Preliminary Results

Table 6 presents the results of the PO portion of the EPQ. The Active Learning subscale earned a score of M = 4.3, SD ± 0.9, in the opinion of the students. The highest-rated question from this subscale was the question related to learning through the teacher’s comments during the simulation and before and after it (question five) (M = 4.5, SD ± 0.7). The question with the lowest rating among the respondents concerned receiving guidance at the appropriate time (for the student) during simulations (question six) (M = 4.1, SD ± 1.0). The second-to-lowest rated item was the question related to discussing the student’s ideas and concepts with the instructor during the simulation (question eight) (M = 4.1, SD ± 1.0). When analyzing the results for the Collaboration subscale of the PO section of the presented tool, it was observed that the highest rating was given by the respondents to question 12 (M = 4.6, SD ± 0.6), which concerned cooperation among the team caring for the patient in a specific clinical situation (Table 2). Question 11 was also highly rated (M = 4.5, SD ± 0.8), which concerned collaboration among participants in the scenario (Table 2). The respondents gave similar assessments for both the Diverse Learning Methods subscale (items 13 and 14, M = 4.2, SD ± 0.9) and the Expectations subscale (items 15 and 16, M = 4.4, SD ± 0.9) (Table 6).

Table 6 also presents the results of the IO section of the EPQ. The validity of the Active Learning subscale features was rated by the students at M = 4.3, SD ± 0.9. As in the case of the PO section, question five in this section of the scale concerned students receiving commentary from the instructor (M = 4.4, SD ± 0.9) and was rated highest by the respondents. The students felt it was important to receive commentary from the instructor before, during, and after high-fidelity simulation classes. On the other hand, the statement that time spent on simulations was more productive (question ten) had the lowest rating (M = 4.1, SD ± 1.0). In the Collaboration subscale, the question on working together with other students (question 11) (M = 4.4, SD ± 0.8) and the question on collaborating in a given clinical situation (question 12) had very similar ratings (Table 2). It was the students’ feeling that the aspect of working with other students and the opportunity for collaborating on patient care with a specific clinical unit were important.

### 3.2. Correlations between Selected Socio-Demographic Variables and the EPQ Scale

When analyzing results for specific points on the scale and subscales of the EPQ in terms of the socio-demographic variables of gender, place of residence, and year of studies, it can be seen that these variables produced significantly diverse results. The results for the scale regarding collaboration in the EPQ questionnaire (Table 7) show a statistically significant differentiation in the points obtained in terms of year of studies (*p* = 0.049). The second year of studies (9.22) demonstrated a significantly higher result in this subscale than did the third year of studies (8.75), and the differentiation of results for the third year was considerably higher (20%). There was no statistically significant difference in the other socio-demographic variables.

Table 8 presents a summary of the results for the EPQ-IO section of the questionnaire. The analysis indicated a significant difference between the respondents in their second year of studies compared to the third-year students. The second-year students demonstrated a higher average (69.7) than did the third-year students (66.3) (*p* = 0.003). The greatest diffusion of intra-group results was recorded in the male group (16%).

## 4. Discussion

Simulation is not only a mode of teaching or a method based on scientific evidence, meant to help students better recognize and understand, in practice, knowledge already acquired; it also significantly supports the development of teamwork [40,41,42,43]. It is a tool by means of which it is possible to assess medical students’ skills and obtain their feedback and opinions on the use, for example, of best educational practices during simulation exercises. This study was the first study in Poland on the validation and adaptation of the EPQ. The Cronbach’s alpha reliability coefficients for this instrument were 0.90 for the EPQ-PO and 0.93 for the EPQ-IO. In addition to Poland, Spain is another European country where the presented scale has been successfully validated. The Cronbach’s alpha reliability coefficients were 0.894 for the EPQ-PO section and 0.0915 for the EPQ-IO section. The Cronbach’s alpha coefficients for the subscales Collaboration, Diverse Learning Methods, and High Expectations in the PO section were 0.860, 0.762, and 0.769, respectively; for the EPQ-IO section, the coefficients were 0.891, 0.832, 0.832, and 0.836, respectively [24]. Another European country where the EPQ scale has obtained very good reliability factor results is Norway. The Cronbach’s alpha coefficients were 0.82 for the EPQ-PO scale and 0.88 for the EPQ-OI scale [25]. The Portuguese version of the EPQ was also characterized by very good parameters for the Cronbach’s alpha reliability coefficient, ranging from 0.70 to 0.86; for the whole instrument, it was 0.90 [28]. Satisfactory results for the Cronbach’s alpha coefficient were also obtained in a study by Franklin et al. on a group of 2200 novice nurses in the southern United States. They obtained an overall EPQ result of 0.95, while the individual subscales ranged from 0.88 to 0.93 [23]. Hong Kong has also succeeded in achieving a very good reliability coefficient for the instrument being studied. A study obtained 0.91 for the EPQ-IO and 0.87 for the EPQ-PO [23]. Efforts have been made in Turkey to determine psychometric values for this scale. The Cronbach’s alpha in the Turkish study ranged from 0.60 to 0.86 [27]. These values are similar to the original scale’s test [16], where the Cronbach’s alpha coefficients were 0.86 for EPQ-PO and 0.91 for EPQ-IO, indicating the high reliability of the instrument and that it can be successfully used in scientific research. CFA results were presented in all the studies listed below, along with their components (CFI, TLI, NFI, AGFI, and others) and results for EFA, KMO coefficients, and Bartlett’s test of sphericity, which involves obtaining empirical evidence for the validity of the construct. Research conducted in Spain and in Norway presented calculations for the CFA in which a four-factor model was proposed, as in the original version of the EPQ [24,25]. In addition, according to the available data in the Spanish EPQ [24] study, all items in the presented model obtained values above 0.50 and were included in the scale. A study conducted in Portugal showed sample fit data determined by the KMO test that equaled 0.81 and Bartlett’s test of sphericity resulted in a value <0.001. Furthermore, a five-component model was proposed, where all items obtained values higher than 0.52. The model accounted for 72% of the variance. Due to certain cultural variations, the authors of the study highlighted cultural differences when translating this scale. In items 8 and 16, the word “instructor” was replaced by “teacher” because, in both Brazil and Portugal, the teacher embodies the role of instructor and of facilitator, while in the United States, these roles are performed by different people [28]. The American study conducted by Franklin et al. presented CFA results where CFI, TLI, NFI, and AGFI exceeded typical acceptance thresholds. The EFA model, however, which would have helped to better adjust the model, was not present [23]. In Hong Kong, the KMO coefficients were very good and suitable for the EPQ-PO and EPQ-IO scales (χ2 = 1456.05; df = 120; *p* ≤ 0.001; χ2 = 2076.87; df = 120; *p* ≤ 0.001). Furthermore, the results for Bartlett’s test of sphericity were 0.87 for the EPQ-PO and 0.91 for the EPQ-IO [26]. In the Turkish study [27], a three-factor model was presented where all items achieved a value of 0.520. This model accounted for 62.50% of the total variance; the first factor had the largest share (40.41%). As in the Spanish study, all items achieved values higher than 0.50 and were therefore not excluded from the scale [24].

When reviewing studies using the EPQ scale, it is worth giving attention to the study by Olaussen et al. [7], which, using the scale under discussion with a group of 187 nursing students, attempted to identify the best elements in the simulation scenario for influencing student satisfaction and confidence in the simulation-based learning process. The mean value for the EPQ scale was M = 4.5, SD ± 0.34. The highest-rated subscale was the Collaboration subscale, which in the opinion of the students received a value of M = 4.90, SD ± 0.26, for the EPQ-PO section and of M = 4.68, SD ± 0.55, for the EPQ-IO section. For its part, the relation between the Active Learning subscale of the EPQ and student satisfaction proved to be statistically significant. In the opinion of the researchers, this could be related to the fact that active learning may be the parent variable; that is, the most significant for students [7].

In their study, Lubbers and Rossman attempted to answer the research question as to the effectiveness of indirect-fidelity simulation and increased student satisfaction and self-confidence after such classes [44]. In a group of sixty-one students, they assessed the best educational practices used during simulation lessons. In the opinion of the students, the highest score was found for the Collaboration subscale (M = 4.64, SD ± 0.45). The study participants gave positive evaluations for experiences of working in a group with others and learning from them; this was expressed by the assessment that “I had the opportunity to work with other students during the simulation” (M = 4.64, SD ± 0.48) and “During the simulation other students and I had to work together in a given clinical situation” (M = 4.64, SD ± 0.52) [44]. In ranking the Polish results, the Collaboration scale—for both the EPQ-PO and EPQ-IO—obtained the highest marks.

In a study conducted by Y. Zhu et al. [19], the EPQ scale was also used to evaluate the application of educational best practice during simulation activities. The study included 90 students from a Chinese university; the participants were persons employed in medical centers as registered nurses (RNs). The values for the individual subscales were as follows: Active Learning, M = 4.15, SD ± 0.46; Collaboration, M = 4.32, SD ± 0.50; Diverse Learning Methods, M = 4.34, SD ± 0.51; and High Expectations, M = 4.35, SD ± 0.48. Interestingly, persons who had greater work experience (more than three years) rated the use of active learning methods higher (M = 4.25, SD ± 0.42) than those with less than three years of experience (M = 4.02, SD ± 0.49). Furthermore, in the case of the Collaboration subscale, it was noted that persons with greater work experience (M = 4.42, SD ± 0.47) rated the collaboration element higher than students just starting work (M = 4.18, SD ± 0.53).

The Active Learning subscale was rated by students in all of the studies as a significant element of simulation-based learning that places the student at the center of instruction. It should be remembered that classes are to be designed taking the needs and opinions of students into consideration. Collaboration is a key element in the nursing profession because the nurse works not only with the patient and his/her family but also forms part of a therapeutic team. In the Polish study, the students also valued the role of collaboration with other students during the implementation of scenarios, as well as in making joint decisions concerning patient care in a given clinical situation.

When analyzing the results for the correlation of the EPQ scale with the presented socio-demographic variables in light of the available literature, an alignment can be noted between the Polish results and a study conducted by Zapko et al. [45]. The Collaboration subscales in both the PO and IO sections had statistically diverse results. The second year of studies demonstrated significantly higher results for this subscale as compared to the third year (*p* = 0.049) for the EPQ-PO section. Working with a group of 461 Saudi nursing students, Albagawi et al. attempted to identify correlating variables and to define competencies in simulation-based education. Year of studies here also proved to be a variable significantly influencing the differentiation of the results for the EPQ scale (*p* = 0.01) [46]. Khasawneh et al., in a study involving 370 nursing students, demonstrated that educational practices measured with the use of the EPQ scale were fully used by students during simulation classes. Moreover, in aligning them with variables such as satisfaction with the learning process, it was apparent that each of the subscales was correlated with the indicated variable (*p* = 0.001) [29]. This thus proves the effectiveness of using the NLN Jeffries theory simulation framework and shows researchers how to proceed when assessing and evaluating simulations using the EPQ scale.

To sum up, it is worthwhile to implement simulations in the teaching system along with best educational practices; i.e., active learning, collaboration, and diverse learning methods. They give students the opportunity to obtain and improve such skills as communication in a group and making joint decisions during the comprehensive care of patients in various states of health.

Moreover, by standardizing appropriate tools for assessing these practices, researchers can acquire the necessary knowledge and skills for creating, implementing, and conducting simulation classes of the highest possible quality [47]. The feedback received through the presented tool will allow medical simulation education to be organized at the highest possible level. Educators responsible for the organization of teaching will be able to use the results of the EPQ scale to ensure the optimal quality of teaching. Referring to students’ opinions in the learning process is an attribute of modern education based on a learning system that significantly increases learners’ involvement in and responsibility for results (Valiga, 2012). In addition, the skills acquired in the classroom will translate directly into the quality of care provided and, thus, into patient safety [48]. The tool in question can also be used in the postgraduate education of medical staff (e.g., post-hospital medical simulation centers) for the professional development of nurses and other health professions [48].

Moreover, the use of the same instruments, making it possible to assess simulations according to the same criteria at the national and international levels, increases their comparability and provides scientific evidence for their continued use and development. The students in the Polish study gave very high ratings to the possibility of collaboration with other students in carrying out scenarios, as well as to joint decision-making regarding given clinical situations of patients. The respondents’ opinions on the best educational techniques were statistically significantly related to their year of study. The second-year students placed more importance on cooperation among simulation participants, joint decision-making, action, and evaluation of results. Moreover, team learning provides opportunities for learning among students with different levels of experience or knowledge, as well as interdisciplinary cooperation.

### Limitations

Due to the presence in the EPQ of two items in the Collaboration, Diverse Learning Methods, and High Expectations subscales, the Cronbach’s alpha coefficient, the value of which depends on the number of items in a given scale or subscale, was not presented [32]. The authors of this study found that, for the best results for scale reliability, it would be appropriate to present the Cronbach’s alpha coefficient result for the entire instrument.

## 5. Conclusions

The results presented provide empirical evidence supporting the reliability and accuracy of the construct of the Polish version of the EPQ in nursing education. Both the Cronbach’s alpha coefficient and the model fit indices in the CFA and EFA proved high enough for the tool to be successfully used in scientific research and in the optimization of the education process using high-fidelity simulation. In conclusion, the translated scales reached at least acceptable values for individual reliability parameters and the instrument reliability of the original English-language version.

## Figures and Tables

**Table 1:** Demographic profile of participants (N = 361).

Sex	Number	%
Female	348	96.4%
Male	13	3.6%
Year of Studies		
Second	184	51%
Third	177	49%
School	Number	%
University	127	35.18%
Vocational Higher Learning	234	64.82%

**Table 2:** Analysis by individual items of the EPQ scale.

	EPQ-PO						EPQ-IO					
	M	SD	Item-Total Correlation	c25	c50	c75	M	SD	Item-Total Correlation	c25	c50	c75
Item 1	4.4	0.9	0.51	4.0	5.0	5.0	4.3	0.9	0.61	4.0	4.0	5.0
Item 2	4.3	0.8	0.45	4.0	4.0	5.0	4.2	0.9	0.63	4.0	4.0	5.0
Item 3	4.4	0.7	0.50	4.0	4.0	5.0	4.2	0.9	0.68	4.0	4.0	5.0
Item 4	4.2	0.9	0.61	4.0	4.0	5.0	4.3	0.8	0.66	4.0	4.0	5.0
Item 5	4.5	0.7	0.59	4.0	5.0	5.0	4.4	0.9	0.62	4.0	5.0	5.0
Item 6	4.1	1.0	0.55	4.0	4.0	5.0	4.3	0.8	0.60	4.0	4.0	5.0
Item 7	4.4	0.8	0.60	4.0	5.0	5.0	4.3	0.9	0.61	4.0	5.0	5.0
Item 8	4.1	1.0	0.56	4.0	4.0	5.0	4.2	0.9	0.62	4.0	4.0	5.0
Item 9	4.3	0.9	0.61	4.0	4.0	5.0	4.2	0.9	0.69	4.0	4.0	5.0
Item 10	4.2	1.0	0.60	4.0	4.0	5.0	4.1	1.0	0.63	4.0	4.0	5.0
Item 11	4.5	0.8	0.52	4.0	5.0	5.0	4.4	0.8	0.62	4.0	5.0	5.0
Item 12	4.6	0.6	0.47	4.0	5.0	5.0	4.4	0.8	0.66	4.0	5.0	5.0
Item 13	4.3	0.9	0.56	4.0	4.0	5.0	4.2	0.9	0.69	4.0	4.0	5.0
Item 14	4.2	0.9	0.55	4.0	4.0	5.0	4.2	0.9	0.65	4.0	4.0	5.0
Item 15	4.4	0.8	0.65	4.0	5.0	5.0	4.4	0.8	0.69	4.0	5.0	5.0
Item 16	4.4	0.9	0.63	4.0	5.0	5.0	4.4	0.8	0.68	4.0	5.0	5.0

M—median, SD—standard deviation, C25, 50, 75—percentiles.

**Table 3:** CFA and EFA fit statistics.

	EPQ-PO		EPQ-IO	
	CFA	EFA	CFA	EFA
RMSEA	0.117	0.118	0.125	0.126
90% CI	0.108; 0.127	0.109; 0.128	0.116; 0.134	0.116; 0.135
CFI	0.768	0.768	0.811	0.811
TLI	0.733	0.733	0.782	0.782
NFI	0.733	0.733	0.784	0.784
WRMR	0.059	0.059	0.058	0.058
SRMR	0.08	0.08	0.073	0.073
Chi square	574.6	574.6	635.9	635.9
*p*-Value	0.0001	0.0001	0.0001	0.0001

**Table 4:** Kaiser–Meyer–Olkin (KMO) sample adequacy test.

KMO and Bartlett’s Tests		
Kaiser–Meyer–Olkin measure of sampling adequacy Kaiser–Meyer–Olkin measure of sampling adequacy EPQ-IO		0.90 0.93
Bartlett’s test of sphericity EPQ-PO	Approx. Chi-Square	2104.67
	df	
	Sig.	
Bartlett’s test of sphericity EPQ-IO	Approx. Chi-Square	2875.62
	df	
	Sig.	

**Table 5:** Table factor loading for EPQ.

	EPQ-PO				EPQ-IO			
	F1	F2	F3	F4	F1	F2	F3	F4
Item 1	0.719						0.655	
Item 2	0.589						0.638	
Item 3	0.697						0.434	
Item 4				0.381		0.327	0.534	
Item 5	0.583						0.554	
Item 6		0.354		0.477		0.479		
Item 7	0.621					0.715		
Item 8		0.308		0.358		0.848		
Item 9				0.509		0.492		
Item 10		0.488			0.642			
Item 11	0.815				0.574			0.542
Item 12	0.651				0.606			
Item 13		0.826			0.898			
Item 14		0.898			0.837			
Item 15			0.782		0.655			
Item 16			0.652		0.589			

**Table 6:** Results for the EPQ PO and EPQ IO subscales.

	EPQ PO		EPQ IO	
	M	SD	M	SD
Active Learning	4.3	0.9	4.3	0.9
Collaboration	4.5	0.9	4.5	0.8
Diverse Learning Methods	4.2	0.9	4.2	0.9
Expectations	4.4	0.9	4.4	0.8
Total	4.3	0.9	4.3	0.8

**Table 7:** Numerical characteristics of results for the EPQ PO questionnaire Collaboration subscale in terms of socio-demographic variables.

EPQ PO Collaboration		M	SD	Min	Max	C25	C50	C75	V	*p*
Age	21 and above	8.89	1.65	2	10	8.0	9	10	19	0.565
	Under 21	9.15	1.11	5	10	9.0	9	10	12	
Gender	Female	8.99	1.49	2	10	8.0	9	10	17	0.723
	Male	8.92	1.44	5	10	8.0	9	10	16	
Place of residence	Urban	8.85	1.63	2	10	8.0	9	10	18	0.334
	Rural	9.05	1.42	2	10	8.5	9	10	16	
Year of studies	Second	9.22	1.08	5	10	9.0	9	10	12	0.049 *
	Third	8.75	1.78	2	10	8.0	9	10	20	

*—statistical significance α = 0.05.

**Table 8:** Numerical characteristics of results for EPQ-IO subscale in terms of socio-demographic variables.

EPQ IO		M	SD	Min	Max	C25	C50	C75	V	*p*
Age	21 and above	67.2	10.39	32	80	61.5	66.0	71	15	0.092 *
	Under 21	69.5	8.45	33	80	65.0	68.0	72	12	
Gender	Female	68.1	9.77	32	80	63.0	67.0	72	14	0.444
	Male	66.2	10.46	51	80	59.0	60.6	69	16	
Place of residence	Urban	67.0	10.34	36	80	62.0	65.0	71	15	0.217
	Rural	68.5	9.52	32	80	64.0	67.0	72	14	
Year of studies	Second	69.7	8.52	33	80	65.0	69.0	73	12	0.003 *
	Third	66.3	10.70	32	80	60.0	65.0	70	16	

*—statistical significance α = 0.05.

## Data Availability

No new data were created or analyzed in this study. Data sharing is not applicable for this article.

## References

[1] Gaba D.M. (2004). The future vision of simulation in health care. Qual. Saf. Health Care.

[2] Jeffries P. (2005). A framework for Designing, Implementing, and Evaluating. Nurs. Educ. Perspect..

[3] Lioce L., Lopreiato J., Downing D., Chang T.P., Robertson J.M., Anderson M., Diaz D.A., Spain A.E. (2020). Healthcare Simulation Dictionary.

[4] Zhu F.-F., Wu L.-R. (2016). The effectiveness of a high-fidelity teaching simulation based on an NLN/Jeffries simulation in the nursing education theoretical framework and its influencing factors. Chin. Nurs. Res..

[5] Kim J., Park J.H., Shin S. (2016). Effectiveness of simulation-based nursing education depending on fidelity: A meta-analysis. BMC Med. Educ..

[6] Lavoie P., Cossette S., Pepin J. (2016). Testing nursing students’ clinical judgment in a patient deterioration simulation scenario: Development of a situation awareness instrument. Nurse Educ. Today.

[7] Olaussen C., Heggdal K., Tvedt C.R. (2020). Elements in scenario-based simulation associated with nursing students’ self-confidence and satisfaction: A cross-sectional study. Nurs. Open.

[8] Dyrstad D.N., Storm M. (2017). Interprofessional simulation to improve patient participation in transitional care. Scand. J. Caring Sci..

[9] World Health Organization (2013). Transforming and Scaling up Health Professionals’ Education and Training.

[10] Taplay K., Jack S.M., Baxter P., Eva K., Martin L. (2015). The Process of Adopting and Incorporating Simulation into Undergraduate Nursing Curricula: A Grounded Theory Study. J. Prof. Nurs..

[11] Culha I. (2014). Active learning methods used in nursing education. J. Pedagog. Res..

[12] Akram A.S. (2018). Gap between Theory and Practice in the Nursing Education: The Role of Clinical Setting. JOJ Nurs. Healt. Care.

[13] Raurell-Torredà M., Bonmatí-Tomás A., Lamoglia-Puig M., Zaragoza-García I., Farrés-Tarafa M., Roldán-Merino J., Gómez-Ibáñez R. (2021). Psychometric design, and validation of a tool to assess the medication administration process through simulation in undergraduate nursing students. Nurse Educ. Today.

[14] Pinar G. (2015). The Effects of High-Fidelity Simulation on Nursing Students’ Perceptions and Self-Efficacy of Obstetric Skills. Int. Arch. Nurs. Health Care.

[15] McMahon E., Jimenez F.A., Lawrence K., Victor J., INACSL Standards Committee (2021). Healthcare Simulation Standards of Best PracticeTM Evaluation of Learning and Performance. Clin. Simul. Nurs..

[16] Jeffries P.R., Rizzolo M.A. (2006). Designing and Implementing Models for the Innovative Use of Simulation to Teach Nursing Care of Ill Adults and Children: A National, Multi-Site, Multi-Method Study.

[17] Cowperthwait A. (2020). NLN/Jeffries Simulation Framework for Simulated Participant Methodology. Clin. Simul. Nurs..

[18] Tagliareni E., Forneris S. (2016). Curriculum and Simulation: Are They Related? Curriculum Evaluating the Simulation: Learning Outcomes.

[19] Zhu Y., Wang A., Bai Y., Xu M., Yin H., Gao Q. (2022). Construction, and practice of a comprehensive nursing skills course with simulation in an RN-BSN program in China: A quasi-experimental study. BMC Med. Educ..

[20] Nowakowski M. (2018). Symulacja medyczna w Polsce—Stan aktualny i perspektywy rozwoju. Ogólnopolski Przegląd Medyczny.

[21] So H.Y., Chen P.-P., Wong G.K.C. (2019). Simulation in medical education. J. R. Coll. Physicians Edinburgh..

[22] Carey J.M., Rossler K. (2022). The How When Why of High-Fidelity Simulation. StatPearls.

[23] Franklin A.E., Burns P., Lee C.S. (2014). Psychometric testing on the NLN Student Satisfaction and Self-Confidence in Learning, Simulation Design Scale, and Educational Practices Questionnaire using a sample of pre-licensure novice nurses. Nurse Educ. Today.

[24] Farrés-Tarafa M., Roldán-Merino J., Lorenzo-Seva U., Hurtado-Pardos B., Biurrun-Garrido A., Molina-Raya L., Morera-Pomarede M.-J., Bande D., Raurell-Torredà M., Casas I. (2020). Reliability and validity study of the Spanish adaptation of the “Educational Practices Questionnaire” (EPQ). PLoS ONE.

[25] Reierson I.Å., Sandvik L., Solli H., Haukedal T.A., Husebø S.E. (2020). Psychometric testing of the Norwegian version of the Simulation Design Scale, the Educational Practices Questionnaire and the Student Satisfaction and Self-Confidence in Learning Scale in nursing education. Int. J. Nurs. Stud. Adv..

[26] Gill B.K. (2020). Translation and validation of the traditional Chinese NLN educational practices questionnaire, simulation design scale and student satisfaction and self-confidence in learning. J. Nurs. Educ. Pract..

[27] Unver V., Basak T., Watts P., Gaioso V., Moss J., Tastan S., Iyigun E., Tosun N. (2017). The reliability and validity of three questionnaires: The Student Satisfaction and Self-Confidence in Learning Scale, Simulation Design Scale, and Educational Practices Questionnaire. Contemp. Nurse.

[28] Dos Santos Almeida R.G., Mazzo A., Martins J.C.A., De Souza-Junior V.D., Mendes I.A.C. (2016). Validação para a língua portuguesa do Educational Practices Questionnaire (Student Version). ACTA Paul. Enferm..

[29] Al Khasawneh E., Arulappan J., Natarajan J.R., Raman S., Isac C. (2021). Efficacy of Simulation Using NLN/Jeffries Nursing Education Simulation Framework on Satisfaction and Self-Confidence of Undergraduate Nursing Students in a Middle Eastern Country. SAGE Open Nurs..

[30] Use of NLN Surveys and Reasearch Instruments. https://www.nln.org/education/training/professional-development-programsresearch/tools-and-instruments.

[31] World Health Organization (WHO) (2016). Process of Translation and Adaptation of Instruments. http://www.who.int/substance_abuse/research_tools/translation/en/.

[32] Tavakol M., Dennick R. (2021). Making sense of Cronbach’s alpha. Int. J. Med. Educ..

[33] Mukaka M.M. (2021). Statistics corner: A guide to appropriate use of correlation coefficient in medical research. Malawi Med. J..

[34] DiStefano C., Liu J., Jiang N., Shi D. (2018). Examination of the Weighted Root Mean Square Residual: Evidence for Trustworthiness?. Struct. Equ. Model..

[35] Edwards J.R., Bagozzi R.P. (2020). On the nature and direction of relationships between constructs and measures. Psychol. Methods.

[36] Hair J.F., Black W.C., Babin B.J., Anderson R.E. (2014). Multivariate Data Analysis: Pearson New International Edition.

[37] de Winter J.C.F., Gosling S.D., Potter J. (2016). Comparing the pearson and spearman correlation coefficients across distributions and sample sizes: A tutorial using simulations and empirical data. Psychol. Methods.

[38] Pallant J. (2016). SPSS Survival Manual: A Step-by-Step Guide to Data Analysis Using IBM SPSS.

[39] Tabachnick B.G., Fidell L.S. (2007). Using Multivariate Statistics.

[40] Hustad J.J., Johannesen B., Fossum M., Hovland O.J. (2019). Nursing students’ transfer of learning outcomes from simulation-based training to clinical practice: A focus-group study. BMC Nurs..

[41] Kantonen J., Lloyd R., Mattila J., Kauppila T., Menezes R. (2015). Impact of an ABCDE team triage process combined with public guidance on the division of work in an emergency department. Scand. J. Prim. Health Care.

[42] Oxelmark L., Nordahl Amorøe T., Carlzon L., Rystedt H. (2017). Students’ Understanding of Teamwork and Professional Roles after Interprofessional Simulation-a Qualitative Analysis. Adv. Simul..

[43] NLN Board of Governors (2015). A Vision for Teaching with Simulation.

[44] Lubbers J., Rossman C. (2017). Satisfaction and Self-Confidence with Nursing Clinical Simulation: Novice Learners, Medium-Fidelity, and Community Settings. Nurse Educ. Today.

[45] Zapko K.A., Lou M., Ferranto G., Blasiman R., Shelestak D. (2018). Evaluating best educational practices, student satisfaction, and self- con fi dence in simulation: A descriptive study. Nurse Educ. Today.

[46] Albagawi B.S., Grande R.A.N., Berdida D.J.E., Raguindin S.M., AlAbd A.M.A. (2021). Correlations and predictors of nursing simulation among Saudi students. Nurs. Forum.

[47] Rutherford-Hemming T., Lioce L., Durham C.F. (2015). Implementing the standards of best practice for simulation. Nurse Educ..

[48] Valiga T.M. (2012). Nursing education trends: Future implications and predictions. Nurs. Clin. N. Am..

